# JNK activity modulates postsynaptic scaffold protein SAP102 and kainate receptor dynamics in dendritic spines

**DOI:** 10.1016/j.jbc.2024.107263

**Published:** 2024-04-04

**Authors:** Stella-Amrei Kunde, Bettina Schmerl, Judith von Sivers, Elham Ahmadyar, Taanisha Gupta, Nils Rademacher, Hanna L. Zieger, Sarah A. Shoichet

**Affiliations:** 1 Neuroscience Research Center NWFZ, Charité – Universitätsmedizin Berlin, Freie Universität Berlin and Humboldt-Universität zu Berlin, Berlin, Germany; 2 Picower Institute for Learning and Memory, Massachusetts Institute of Technology, Cambridge, Massachusetts, USA; 3 German Center for Neurodegenerative Diseases (DZNE) Berlin, Berlin, Germany; 4 CNRS, Interdisciplinary Institute for Neuroscience (IINS), UMR 5297, University of Bordeaux, Bordeaux, France

**Keywords:** neuron, synapse, scaffold protein, kainate receptor, JNK, signalling, neurodevelopmental disorders, receptor trafficking

## Abstract

Synapse formation depends on the coordinated expression and regulation of scaffold proteins. The JNK family kinases play a role in scaffold protein regulation, but the nature of this functional interaction in dendritic spines requires further investigation. Here, using a combination of biochemical methods and live-cell imaging strategies, we show that the dynamics of the synaptic scaffold molecule SAP102 are negatively regulated by JNK inhibition, that SAP102 is a direct phosphorylation target of JNK3, and that SAP102 regulation by JNK is restricted to neurons that harbor mature synapses. We further demonstrate that SAP102 and JNK3 cooperate in the regulated trafficking of kainate receptors to the cell membrane. Specifically, we observe that SAP102, JNK3, and the kainate receptor subunit GluK2 exhibit overlapping expression at synaptic sites and that modulating JNK activity influences the surface expression of the kainate receptor subunit GluK2 in a neuronal context. We also show that SAP102 participates in this process in a JNK-dependent fashion. In summary, our data support a model in which JNK-mediated regulation of SAP102 influences the dynamic trafficking of kainate receptors to postsynaptic sites, and thus shed light on common pathophysiological mechanisms underlying the cognitive developmental defects associated with diverse mutations.

Proper network formation relies on the regulated expression of diverse neuronal proteins and also on the coordinated generation and modulation of synaptic connections during development. In excitatory neurons, synaptic function is largely dependent on the integrity of the postsynaptic density (PSD); the molecular composition of the PSD has therefore been studied in considerable depth (for reviews, see *e.g.*, (1, 2)). In addition to neurotransmitter receptors and transsynaptic cell adhesion molecules at the postsynaptic membrane, scaffold proteins play an integral role in defining the functional architecture of the PSD, and their coordinated expression and regulation is critical for proper synapse formation and maintenance. It follows from this that mutations in diverse postsynaptic scaffold proteins have been implicated in neurodevelopmental disorders (see *e.g.*, (3, 4, 5) for reviews).

PSD-95 family MAGUKs are among the most abundant scaffold proteins at the postsynaptic sites of glutamatergic neurons, and they are recognized as central building blocks of the PSD (6). They are critical for coordinating the trafficking and anchoring of glutamate receptors at the postsynaptic membrane (7), and these functions of MAGUKs are especially important during development (8) and in the regulation of synaptic plasticity (9). Among synaptic MAGUKs, SAP102 is recognized for its high expression during early development and critical role in synapse development and maturation (10, 11). In line with an important developmental function of this particular synaptic MAGUK, numerous monogenic forms of developmental delay have been linked to genetic alterations of SAP102 (12, 13, 14, 15, 16) (gene *DLG3*, see also OMIM #300850 MRX90). SAP102 also differs functionally from other synaptic MAGUKs in that it behaves differently in dendritic spines: while PSD-95 is anchored stably at the PSD, SAP102 exhibits a comparatively high mobility into and out of the spines (17), suggesting a unique role for this MAGUK in receptor trafficking to and from synaptic sites. Moreover, it has been shown that the rate with which SAP102 enters and exits the spine can be influenced by posttranslational modifications (18, 19), highlighting that the cellular function of SAP102 can potentially be regulated in response to extracellular signals, which are especially relevant during synapse formation, learning, and excitotoxic stress.

We have previously shown that SAP102 is able to bind to the c-Jun N-terminal kinase (JNK)-3 (20), which is the central nervous system-specific member of the JNK family of mitogen-activated protein (MAP) kinases, that is, the terminal kinases in one branch of the MAP kinase cascade that coordinates cellular responses to diverse external stimuli. JNK3 mutations have been implicated in cognitive and seizure disorders in young children (20, 21), and while JNK family kinases are generally known for their role in the cellular stress response, in neurons, they serve as established regulators of neuronal differentiation and plasticity (for reviews see (22, 23, 24)), and they are capable of phosphorylating both scaffolds and neurotransmitter receptor subunits (25, 26). Here, we have investigated the functional links between the scaffold protein SAP102 and the neuronal kinase JNK3, and we demonstrate that the behavior of SAP102, in particular its mobility into and out of dendritic spines, is influenced by JNK signaling. We next demonstrated that kainate receptor trafficking can likewise be influenced by JNK regulation, and we showed that SAP102 forms a specific complex with JNK3 and the kainate receptor subunit GluK2; we thus provide insights into how these three proteins participate in a common molecular cascade that is disturbed in multiple monogenic neurodevelopmental disorders.

## Results

### SAP102 mobility is modulated by JNK activity

**Figure 1:**
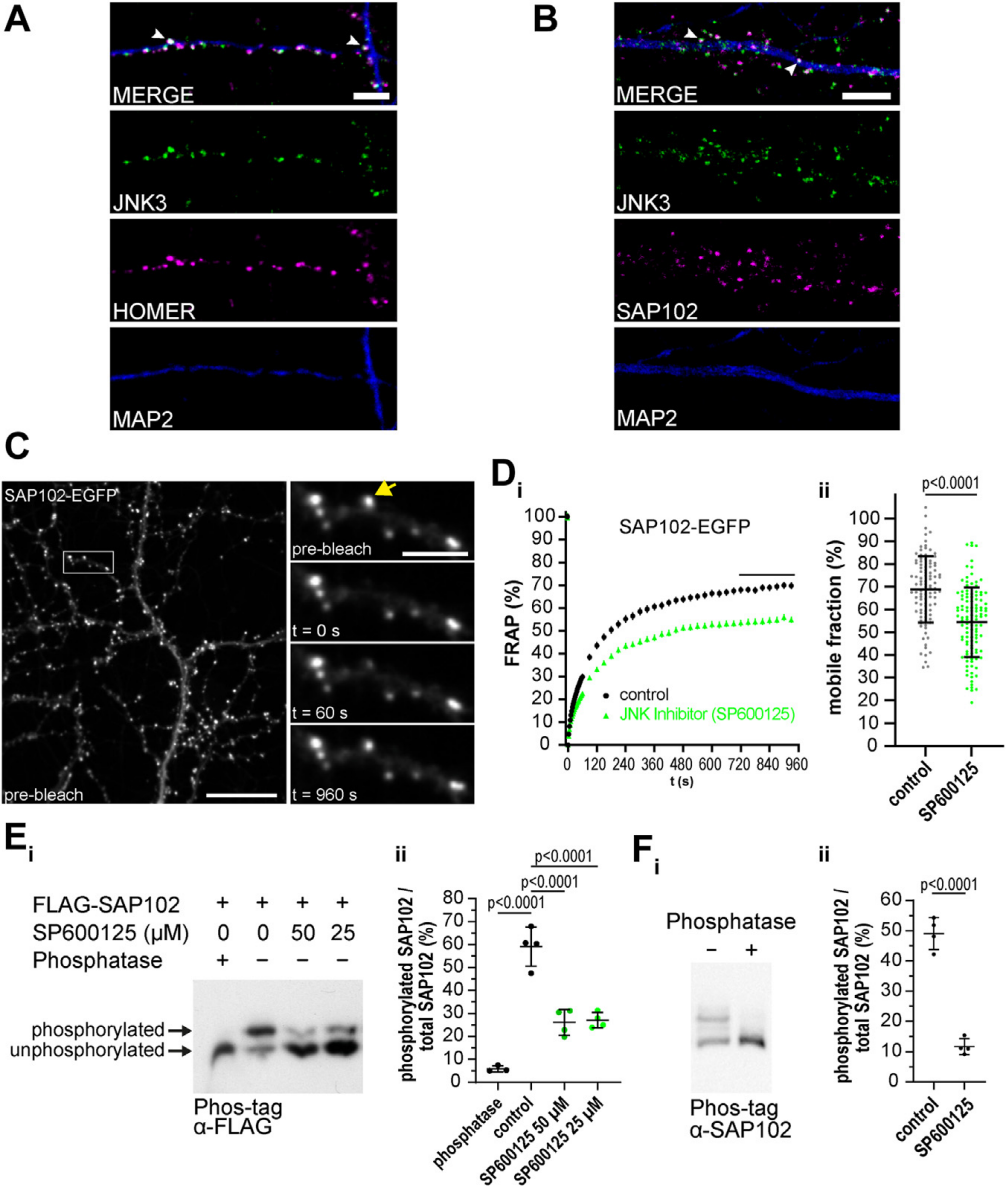
**SAP102 mobility is modulated by JNK activity.***A*, coimmunofluorescence of JNK3 (*green*, Alexa488) with synaptic marker protein Homer (*magenta*, Alexa568) in MAP2-positive dendrites (*blue*, Alexa405) of primary rat hippocampal neurons (DIV22), analyzed by confocal microscopy. The scale bar represents 5 μm. *B*, coimmunofluorescence of endogenous JNK3 (*green*, Alexa488) with SAP102 (*magenta*, Alexa568) in MAP2-positive dendrites (*blue*, Alexa405) of primary rat hippocampal neurons (DIV22), analyzed by confocal microscopy. The scale bar represents 5 μm. Immunofluorescence images (single *z*-stacks) are representative examples from three independent experiments. *C*, live-cell imaging of SAP102-EGFP, following lentivirus-mediated gene delivery in primary rat hippocampal neurons (DIV23), using spinning disc microscopy for FRAP experiments: representative images of the same ROI with SAP102-EGFP in spines before photo-bleaching (prebleach), and at t = 0 s, t = 60 s, and t = 960 s after photo-bleaching. The scale bar represents 20 μm (*overview*) or 5 μm (*zoom*). *D*, *i*: FRAP experiments of SAP102-EGFP in spines showing control (*black circle*) *versus* JNK inhibitor SP600125-treated (*green triangle*) rat primary hippocampal neurons (25 μM SP600125, 2 h). Data are background subtracted, normalized, and expressed as mean ± SEM, with n = 101 to 113 analyzed spines from 11 images per condition from three independent neuronal cultures. *ii*, the mobile fraction was calculated as the mean of the last eight FRAP values of SAP102-EGFP for all synapses analyzed in all ROIs in both conditions (see *black bar* in (*i*)). Individual datapoints as well as the mean ± SD are shown. Data passed normality test (D’Agostino and Pearson test). Statistical significance was determined by performing unpaired two-tailed *t* test. *E*, *i*: FLAG-SAP102 is phosphorylated in CHL cells. Phosphorylated proteins are separated from unphosphorylated proteins using Phos-tag-SDS-PAGE gels and analyzed by Western blot (α-FLAG). Phosphatase treatment shows unphosphorylated FLAG-SAP102 as control. Treatment of cells with JNK inhibitor (SP600125: 50 μM/25 μM) results in a decrease of phosphorylated SAP102 protein. *ii*, quantification of phosphorylated FLAG-SAP102/total FLAG-SAP102 where total FLAG-SAP102 in Phos-tag experiments is the sum of phosphorylated and unphosphorylated FLAG-SAP102. Data represent mean ratio ± SD from four independent experiments. Data passed normality test (Shapiro–Wilk) and statistical significance was determined by performing one-way ANOVA/Dunnett’s multiple comparison test. *F*, *i*: endogenous SAP102 is phosphorylated in primary rat hippocampal neurons (DIV24); phosphatase treatment of cell lysate serves as control in Phos-tag/SDS-PAGE experiments (detection with α-SAP102). *ii*, quantification of phosphorylated endogenous SAP102/total SAP102 ratio from (*i*). Data represent mean ratio ± SD from four independent experiments. Statistical significance was determined by performing unpaired two-tailed *t* test. CHL, Chinese hamster lung; FRAP, fluorescence recovery after photobleaching.

In immunofluorescence (IF) experiments, we observe colocalization of endogenous JNK3 with the postsynaptic marker protein Homer in dendritic spines of mature primary neurons (Fig. 1*A*), confirming the presence of endogenous JNK3 at postsynaptic sites. We have previously shown that JNK3 binds to the postsynaptic scaffold protein SAP102 (20), and we observe overlapping expression of these two proteins in dendritic spines (Fig. 1*B*). In order to explore the consequences of the JNK–SAP102 interaction at synaptic sites, we generated a recombinant SAP102 protein tagged with enhanced green fluorescent protein (EGFP) for analysis in living neurons.

SAP102 is a highly mobile scaffold protein, capable of moving rapidly into and out of dendritic spines (17). Using fluorescence recovery after photobleaching (FRAP) of SAP102-EGFP in spines of rat hippocampal neurons (Fig. 1*C*), we explored the effects of JNK modulation on SAP102 mobility. Following application of the JNK inhibitor SP600125, the mobile fraction of SAP102 is substantially reduced (Fig. 1*Di* with quantification of the difference in the mobile fraction in Fig 1*Dii*), suggesting a functional link between SAP102 and JNK activity that we investigated in subsequent experiments.

We first explored the idea that this functional link might reflect a direct JNK-mediated phosphorylation of SAP102. Using the Phos-tag system (27), we analyzed the phosphorylation status of SAP102: following overexpression in heterologous cells, we observed a distinct single phosphorylation of the overexpressed FLAG-tagged SAP102 (Fig. 1*Ei* upper band). The phosphorylated SAP102 protein (upper band) was completely absent after treatment with protein phosphatase, and the ratio of phosphorylated to unphosphorylated SAP102 was reduced in a dose-dependent fashion in response to treatment with a JNK inhibitor (see quantification of this reduction in Fig. 1*Eii*), suggesting that the observed SAP102 phosphorylation in heterologous cells is indeed JNK-dependent. In line with a basal physiological phosphorylation of SAP102 that could potentially be attributed to the presence of interacting JNK proteins in neurons, Phos-tag analysis of endogenous SAP102 in neuronal lysates suggests that a substantial portion of the endogenous SAP102 protein is phosphorylated in mature cultured primary neurons (Fig. 1*F*). Together, these results indicate that SAP102 mobility can be modulated by JNK activation status, and they provide evidence in support of a direct JNK-mediated phosphorylation of SAP102 that may influence its behavior.

### SAP102 is phosphorylated at position S368 by JNK

**Figure 2:**
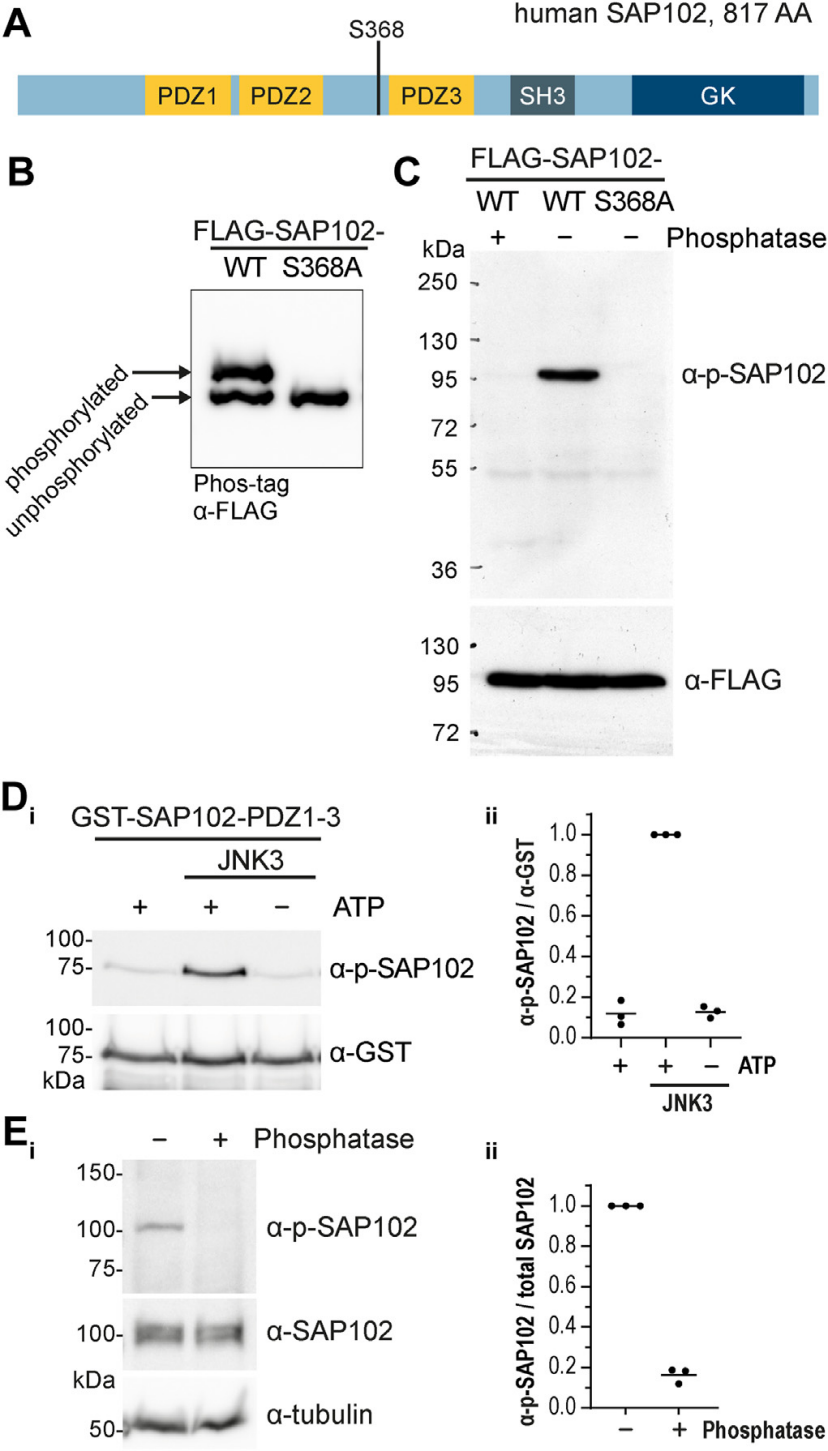
**SAP102 is phosphorylated at S368 by JNK.***A*, schematic overview of the human SAP102 MAGUK protein (UniProt #Q92796, 817 AA) with its annotated domains (PDZ1/2/3, SH3, and GK, drawn to scale) and phosphorylation site S368. *B*, FLAG-SAP102 is phosphorylated at position S368 in CHL cells. Phosphorylated proteins are separated from unphosphorylated proteins using Phos-tag-SDS-PAGE gels and analyzed by Western blot (α-FLAG). Phospho-deficient point mutation at position S368 of FLAG-SAP102 showed only one band (n > 5 independent experiments). *C*, FLAG-SAP102-WT and FLAG-SAP102 with phospho-deficient mutation S368A (FLAG-SAP102-S368A) were transfected in CHL cells and analyzed by Western blot for phosphorylation using the α-p-SAP102 antibody (raised against a short peptide harboring a phosphorylated S368). The dephosphorylated FLAG-SAP102 (phosphatase treatment) and the phospho-deficient mutation (FLAG-SAP102-S368A) are not detected by α-p-SAP102 (n > 5 independent experiments). α-FLAG detection serves as expression control of FLAG-SAP102 constructs. *D*, *i*: *in vitro* kinase assay of bacterially expressed and purified GST-SAP102-PDZ1-3 protein with active kinase JNK3, followed by Western blot. α-p-SAP102 detects phosphorylated SAP102, α-GST serves as loading control for the GST substrate. *ii*, quantification of phosphorylated GST-SAP102-PDZ1-3/total GST-SAP102-PDZ1-3 from (*i*). Data represent mean ratio from three independent experiments. *E*, *i*: SAP102 is phosphorylated in primary rat hippocampal neurons. Lysates (DIV24) were analyzed by Western blot with the antibodies indicated; α-tubulin served as loading control. Phosphatase treatment of the lysate served as control for specificity of phosphoprotein detection. *ii*, quantification of neuronal phosphorylated SAP102/total SAP102 from (*i*). Data represent mean ratio from three independent experiments. CHL, Chinese hamster lung; GST, glutathione-S-transferase.

Next, we explored the putative phosphorylation of SAP102 by JNK in more detail. We analyzed the SAP102 amino acid sequence for putative MAPK/JNK phosphorylation motifs and focused on the proline-directed serine at amino acid 368 of SAP102 that lies in close proximity to the predicted JNK docking site (28) in a disordered region of the linker between the second PDZ domain and the MAGUK module PDZ3-SH3-GK of SAP102 (see scheme in Fig. 2*A*). Upon mutation of this serine to alanine, phosphorylation of the tagged SAP102 (as assessed by Phos-tag assay following expression in heterologous cells) was no longer detectable (see Fig. 2*B*), indicating that indeed the observed phosphorylation of overexpressed SAP102 reflects phosphorylation at this site. To study this phosphorylation in detail, we raised phospho-specific antibodies against a peptide in which the serine at position 368 was phosphorylated. As expected, the affinity-purified antibody (α-p-SAP102) recognized the phosphorylated form of SAP102: in lysates from heterologous cells overexpressing FLAG-tagged WT SAP102, we observed a single band at the expected size that was not present in lysates that had been treated with phosphatase (Fig. 2*C*). Importantly, the α-p-SAP102 did not recognize the overexpressed human SAP102 with S368A mutation (Fig. 2*C* lane 3), further confirming its specificity. This antibody was subsequently used to assess the phosphorylation of SAP102 at serine 368 by JNK3: in *in vitro* nonradioactive kinase assays, we observed a clear kinase-dependent accumulation of phosphorylated SAP102 that was not observed in control samples lacking either the kinase or the ATP (Fig. 2*D*). Finally, we confirmed that our phospho-specific SAP102 antibody specifically recognized a protein in primary cultured neurons of the expected size (approximately 100 kDa) and that phosphatase treatment of these cultured neurons resulted in loss of the signal, while total SAP102 could be detected comparably in both treated and untreated samples *via* Western blot (WB) with the well-characterized commercially available mouse monoclonal SAP102 antibody (Fig. 2*E*).

### JNK-mediated SAP102 phosphorylation is activity-dependent and developmentally regulated

**Figure 3:**
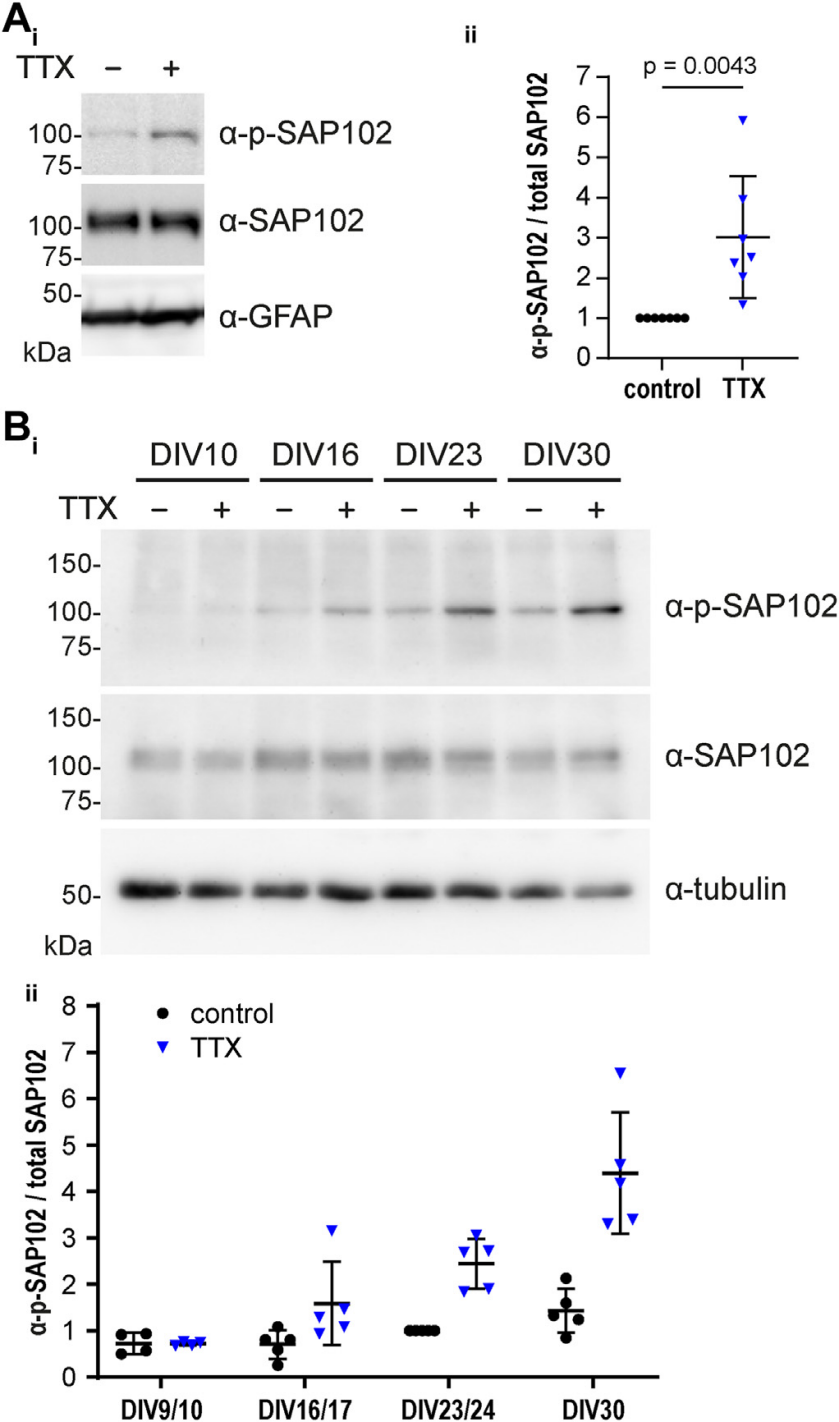
**JNK-mediated SAP102 phosphorylation is activity-dependent and developmentally regulated.***A*, *i*: TTX treatment (2 μM, O/N) of primary rat hippocampal neuron cultures (DIV23) increases SAP102 phosphorylation, as observable by Western blot with the phospho-specific SAP102 antibody (α-p-SAP102). α-SAP102, and α-GFAP served as controls. *ii*, quantification of neuronal p-SAP102/total SAP102 from (*i*), normalized to untreated control. Data represent mean ratio ± SD from seven independent experiments. Statistical significance was determined by performing unpaired two-tailed *t* test. *B*, *i*: SAP102 phosphorylation in primary rat hippocampal neurons at DIV10, DIV16, DIV23, and DIV30 was analyzed by Western blot with or without TTX treatment (2 μM, O/N). TTX-induced phosphorylation of SAP102 increases at later developmental stages (DIV 16, DIV23, and DIV30). α-SAP102 and α-tubulin served as controls. *ii*, quantification of neuronal phosphorylated SAP102/total SAP102 shown in (*i*). Data represent mean ratio ± SD from five independent experiments (exception: for DIV9/10 n = 4). All data were normalized to internal experimental control values for DIV23/24.

We next investigated JNK-dependent phosphorylation of SAP102 in primary hippocampal neurons. In lysates from mature rat hippocampal neurons (DIV23-24), we consistently observed a basal phosphorylation of SAP102 (see Figs. 1*F* and 2*E*). Interestingly, this basal phosphorylation was much more difficult to detect in immature neurons, that is, prior to synaptogenesis (see Fig. 3*Bi*, lane 1, *i.e.*, DIV10 untreated neurons). This led us to hypothesize that JNK-mediated phosphorylation of SAP102 might be important in the regulation of synaptic connections, rather than in early development prior to synapse maturation.

To explore the role of JNK-mediated phosphorylation of SAP102 in such dynamic processes, we induced synaptic upscaling (homeostatic synaptic plasticity) by activity blockade (blocking of Na^2+^ channels with tetrodotoxin [TTX]). Indeed, the phosphorylation of SAP102 at serine 368 strongly increased in mature neurons in response to TTX treatment (Fig. 3*Ai* with quantification in Fig. 3*Aii*; see also Fig. S1*i*), suggesting that JNK might play a role in synaptic activity-dependent regulation of SAP102. In a subsequent set of experiments that aimed to assess this developmental role of activity-induced SAP102 phosphorylation, we observed that this TTX-mediated phosphorylation of SAP102 was indeed developmentally regulated: when rat hippocampal neurons were analyzed at different developmental time points for SAP102 phosphorylation (Fig. 3*B*), we consistently detected significant basal phosphorylation of SAP102 at S368 at time points after DIV15, whereas at earlier time points (DIV9-10), we detected only minimal expression of phosphorylated SAP102 despite significant expression of the unphosphorylated protein (compare lanes 1, 3, 5, and 7 in Fig 3*Bi*; see also quantification in Fig. 3*Bii*). Also relevant in this context: in more mature neurons, the relative phosphorylation of SAP102 at S368 increased when neurons were treated with TTX, whereas there was no clear TTX-induced increase of phosphorylation at DIV9-10. The observed increase in basal phosphorylation during development corresponds to the period after synapse formation, in line with a putative role for SAP102 phosphorylation at synapses. The idea that JNK-mediated regulation of SAP102 is part of the molecular response to changes in synaptic activity is further supported by the fact that we consistently observe a strong increase in phosphorylation during the process of synaptic upscaling, that is, after TTX treatment in mature neurons (see comparative quantitative analysis of SAP102 phosphorylation from multiple time course experiments in Fig. 3*Bii*).

Importantly, we observed this increase of SAP102 phosphorylation following TTX treatment not only in whole-cell lysates from cultured rat hippocampal neurons but also in crude synaptosome fractions (Fig. S1*ii*), suggesting that SAP102 is indeed phosphorylated when present in synapse-enriched fractions, which is in line with a role for JNK-mediated regulation of SAP102 at synaptic sites.

### Neuronal activity blockade and JNK inhibition exert opposing effects on SAP102 mobility

We have clearly shown that SAP102 is phosphorylated by JNK and that this phosphorylation is developmentally regulated and modulated by neuronal activity (Fig. 3). Given that JNK3 promotes SAP102 phosphorylation (Fig. 2*D*) and that JNK inhibition modulates SAP102 mobility (Fig. 1), combined with the fact that SAP102 phosphorylation by JNK is activity-dependent, we hypothesized that SAP102 mobility might likewise be influenced by modulating neuronal activity.

**Figure 4:**
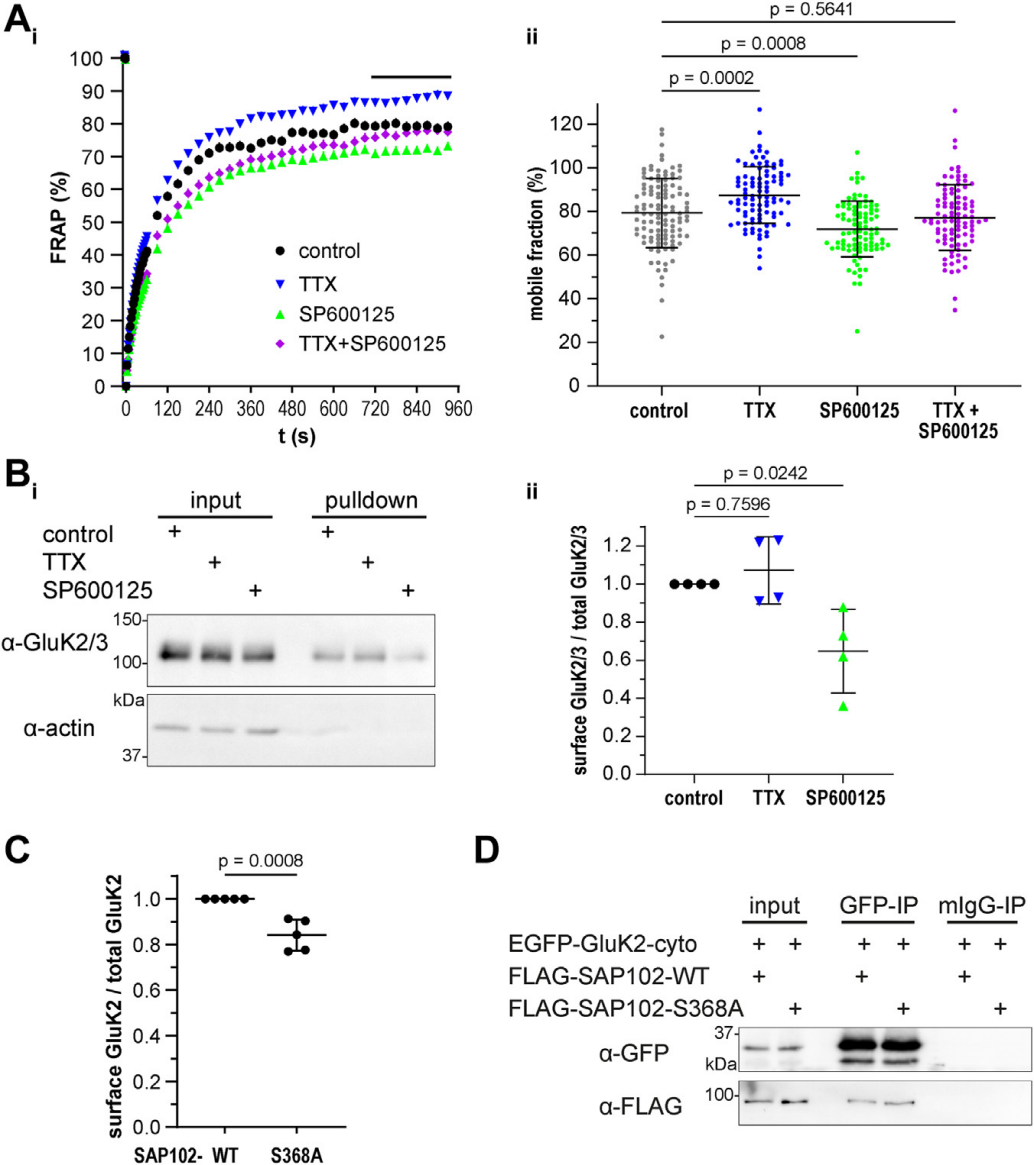
**Both activity blockade and JNK activity promote mobility of SAP102 and GluK2 surface expression.***A*, *i*: FRAP experiments of SAP102-EGFP localized in spines of rat hippocampal neurons (DIV20–DIV23) were performed using live-cell imaging with spinning disc microscopy. Samples were treated with either TTX (2 μM, O/N), JNK inhibitor SP600125 (25 μM, 2 h) or TTX and SP600125 (2 μM TTX O/N and 25 μM SP600125 2 h prior to measurement). Data are background-subtracted, normalized (to the mean of seven prebleach acquisitions (=100%) as well as to the value at t = 0 s (=0%)) and show the mean ± SEM of n = 94 to 112 spines per condition (10–15 spines per image; 8–10 images per condition acquired from three independent neuronal cultures). FRAP data were acquired for 960 s *ii*, the mobile fraction was calculated as the mean of the last eight FRAP values of SAP102-EGFP in each condition (*black bar* in (*i*)). Individual datapoints as well the mean ± SD are shown. Data passed normality test (D’Agostino and Pearson test) and statistical significance was determined by performing one-way ANOVA/Dunnett’s multiple comparison test of all samples compared to control. *B*, *i*: surface biotinylation experiment in rat hippocampal neurons (DIV21) after O/N treatment with either TTX (2 μM) or JNK inhibitor SP600125 (25 μM). Proteins located at the cell surface were biotinylated and after pulldown with streptavidin-Dynabeads, surface expression of GluK2/3 was analyzed by Western blot (comparison of pulldown to input). The cytoplasmic protein actin (α-actin) serves as negative control for the surface biotinylation procedure (as expected, cytosolic proteins are not labeled). *ii*, quantification of surface GluK2/3 (pulldown)/total GluK2/3 (input) from (*i*). Data represent mean ratio ± SD from four independent experiments. Data passed normality test (Shapiro–Wilk test) and statistical significance was determined by performing one-way ANOVA/Dunnett’s multiple comparison test of all samples compared to control. *C*, analysis of GluK2 surface expression (relative to total GluK2 expression) following expression in CHL cells (on-cell Western, OCW) together with WT SAP102 enables comparison of WT and phospho-deficient SAP102 (S368A) with regard to their ability to promote GluK2 surface expression. Data are mean ratios ± SD from five independent experiments; GluK2 surface data was normalized to values obtained for the WT constructs after subtraction of background fluorescence (as an internal experimental control for background fluorescence, we took advantage of the fully internalized GluK2-INT; see Fig. S4). Statistical significance was determined by performing unpaired two-tailed *t* test. *D*, FLAG-tagged SAP102 (either WT or S368A phospho-deficient mutant as indicated) were coexpressed with the EGFP-tagged GluK2 cytosolic region in CHL cells. Following pulldown of the GluK2 C terminus with the GFP antibody, coimmunoprecipitated SAP102 variants were analyzed by Western blot (α-FLAG), which indicated comparable interaction of the two variants with the GluK2 C-terminus. Mouse IgG served as negative control for the pulldown (mIgG-IP). Data shown is a representative example of three independent biological replicates. CHL, Chinese hamster lung; FRAP, fluorescence recovery after photobleaching.

We induced homeostatic plasticity with TTX-mediated activity blockade, and we subsequently analyzed the mobility of SAP102 using FRAP (Fig. 4*A*). The mobile fraction of SAP102-EGFP, based on the average of values obtained for the final eight postbleach values recorded for each synapse, indeed increased after induction of synaptic scaling, whereas it decreased following JNK inhibition. Simultaneous combination of the two treatments resulted in a compensation, yielding a mobility that was comparable to that for SAP102 in the untreated control condition (see Fig. 4*Aii* for quantification and statistics on the mobile fraction comparison). Together, these data indicated that JNK inhibition and TTX-mediated activity blockade have contrasting effects on SAP102 behavior in this context and highlight a putative role for JNK in coordinating the dynamics of the SAP102 function in dendritic spines, perhaps in concert with TTX-induced signal cascades that modulate homeostatic plasticity.

In line with this JNK inhibitor–induced reduction in the mobile fraction, the t_1/2_—which reflects the speed of recovery (half-recovery time)—was similarly affected by JNK modulation (see Fig. S2): This JNK-mediated reduction in the recovery speed was not substantially altered by the additional application of TTX, suggesting that JNK activity may act downstream in homeostatic mechanisms that mediate SAP102 accumulation at synapses.

In summary, these FRAP results show that JNK inhibition hinders SAP102 movement into and out of dendritic spines. Moreover, in contrast to treatments that block activity and result in synaptic upscaling, JNK inhibition reduces the fraction of SAP102 molecules that are mobile. We conclude that JNK activity has a positive regulatory effect on SAP102 mobility and thus may similarly influence SAP102 binding partners.

### SAP102 promotes KAR surface expression through formation of a functional tripartite complex with JNK

SAP102 has been shown to bind directly to glutamate receptors (*e.g.*, NMDARs, kainic acid receptors [KARs], and AMPAR auxiliary proteins). We therefore asked whether SAP102—with the assistance of JNK—might be responsible for the shuttling of such receptors into or out of the spines during synaptic scaling. GluK2 is particularly interesting in the context of JNK regulation because there are similarities between the GluK2 and JNK3 KO mice; moreover, patients with GluK2 or JNK3 mutations have overlapping phenotypic features (20, 21, 29). IF analysis of the three proteins in primary hippocampal neurons fixed at DIV 21 confirmed that they indeed exhibit overlapping localization in dendritic spines (see Fig. S3). We next assessed the surface expression of endogenous GluK2 in surface biotinylation experiments (Fig. 4*B*). Following JNK inhibition in primary rat hippocampal neurons, a strong decrease in GluK2 surface expression was observed, whereas receptor surface expression after synaptic upscaling (TTX treatment) increased (see Fig. 4*Bi* with quantification and statistics in Fig. 4*Bii*). These data are in line with a role for JNK-mediated regulation of the shuttling of kainate receptors to the surface. Together with our previous results demonstrating the JNK-mediated regulation of SAP102 mobility, this result suggests that SAP102 and JNK may work together to coordinate kainate receptor trafficking.

To investigate this hypothesis, we took advantage of an in-cell/on-cell WB assay in heterologous cells, which enabled us to specifically examine the cooperative effects of JNK and SAP102 on kainate receptor subunit surface expression. Following expression of full-length GluK2 together with WT SAP102, we observed that a substantial fraction of overexpressed GluK2 was integrated into the membrane and thus detectable by staining prior to cell permeabilization. For comparison, we took advantage of targeted GluK2 mutants that have been shown to remain internalized (30). For these variants, expression ratios (surface:total) were comparable to ratios for proteins that are entirely cytoplasmic (see Fig. S4); we thus took advantage of this GluK2 variant to reflect our baseline fluorescence for receptor surface expression in subsequent experiments.

We next compared the effects of WT SAP102 with that of SAP102 harboring a point mutation at the JNK phospho-site that precluded positive regulation by JNK (see SAP102 S368A depicted in Fig. 2*A*) on GluK2 surface expression in this assay. Compared to the WT, the SAP102 S368A mutant exhibited a reduced ability to promote the surface expression of the WT GluK2 subunit, suggesting that JNK-mediated phosphorylation of SAP102 indeed facilitates membrane expression of GluK2, as we hypothesized (see Fig. 4*C*). In control experiments, we confirmed that both WT and phospho-deficient SAP102 mutants bound efficiently to GluK2 (see Fig. 4*D*), thereby excluding the possibility that differences in surface expression reflect reduced binding of the GluK2 C terminus to SAP102 PDZ domains. We also tested the effects of JNK inhibition on the surface expression of GluK2 in this assay and observed a trend suggesting that the inhibitor mediates a reduction in the relative surface expression GluK2 (see Fig. S5), in line with our data for the S368A mutant that highlights a JNK-dependent effect on SAP102-mediated GluK2 surface expression.

**Figure 5:**
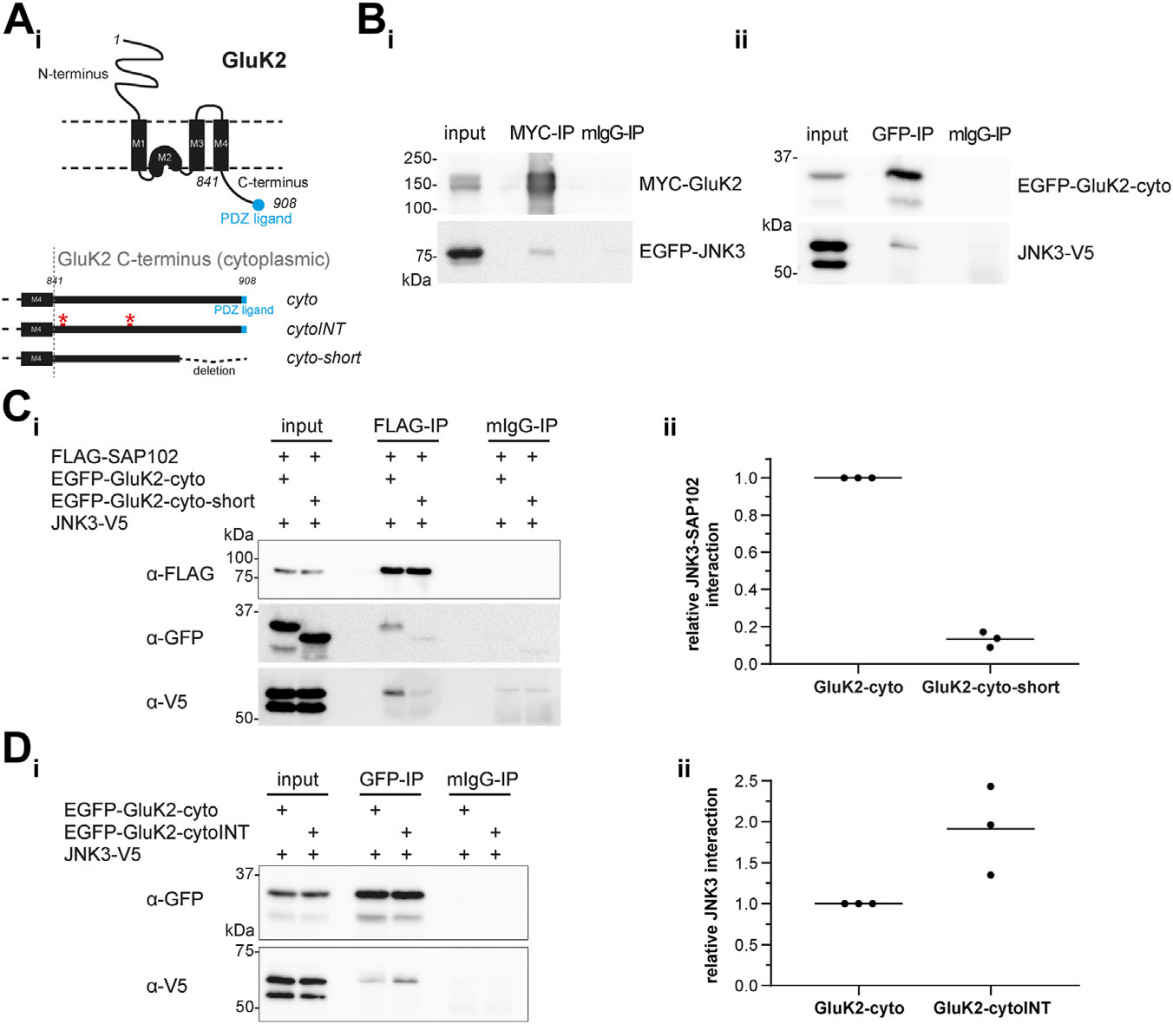
**JNK3–SAP102–GluK2 protein complex.***A*, schematic overview of the full-length kainate receptor subunit GluK2 (UniProt P39087), depicting the extracellular N terminus, the membrane-integrated domains (M1-4), and the intracellular, cytoplasmic C terminus, including the PDZ ligand (*blue*). Below an enlargement of the cytoplasmic GluK2 C terminus is shown (drawn to scale), summarizing the protein variants used in this study, including GluK2-cyto (with the WT PDZ-binding C terminus), GluK2-cytoINT, which harbors mutations at two sites that are important for GluK2 receptor internalization (highlighted in *red*) and GluK2-cyto-short, which reflects the WT C terminus with a 26 AA deletion of the C terminus. *B*–*D*, interactions between of JNK3, GluK2, and SAP102 protein variants were analyzed in coimmunoprecipitation experiments following expression in HEKT cells: proteins were precipitated using the antibodies indicated above, (mIgG served as negative control), and samples were analyzed by Western blot using antibodies as indicated. *B*, *i*: pulldown of overexpressed full-length MYC-GluK2 (MYC-IP) showed the coimmunoprecipitation with EGFP-JNK3 (n = 3). *ii*, pulldown of the overexpressed cytoplasmic GluK2 C terminus (EGFP-GluK2-cyto, GFP-IP) showed coimmunoprecipitation with JNK3-V5 (n = 3). *C*, *i*: the analysis of comparative coimmunoprecipitation experiments of overexpressed FLAG-SAP102 with JNK3-V5 and either EGFP-GluK2-cyto or EGFP-GluK2-cyto-short (deletion of PDZ ligand) indicated an increased tripartite complex formation when the cytoplasmic GluK2-C-terminus was intact (EGFP-Gluk2-cyto). *ii*, quantification of normalized JNK3-SAP102 interaction (GFP/FLAG signal) from three independent experiments; datapoints for individual experiments and their mean is shown. *D*, *i*: pulldown of either the WT GluK2 C terminus (EGFP-GluK2-cyto) or the internalized variant (EGFP-GluK2-cytoINT) with a GFP antibody (GFP-IP) enabled comparison of their ability to bind JNK3 (observable in Western blot of coprecipitated JNK3 with the α-V5 antibodies). *ii*, quantification of normalized JNK3–GluK2 interaction (V5/GFP signal) from three independent experiments (n = 3), datapoints of individual experiments and their mean are shown. HEK, human embryonic kidney.

To further investigate this functional protein complex comprised of GluK2, SAP102, and JNK, we took advantage of coimmunoprecipitation (Co-IP) assays with diverse combinations of recombinant proteins (see constructs depicted in Fig. 5*A*). Initially, we focused on the analysis of protein complexes that included JNK3 and GluK2. After expression of the full-length GluK2 together with JNK3 and pull-down of GluK2, we observed a clear Co-IP of JNK3 (Fig. 5*Bi*). In subsequent experiments, we demonstrated that the GluK2 C-terminal cytoplasmic region (see schematic in Fig. 5*A*) is sufficient to enable binding to JNK3 (see Fig. 5*Bii*). As expected, this cytosolic region, which harbors a PDZ-binding motif at its distal C terminus, also interacts with SAP102 *via* classical ligand–PDZ domain interactions that rely on the most C-terminal residues (see Fig. S6; see also (31, 32, 33)).

Interestingly, the presence of the WT GluK2 cytoplasmic region strengthens the interaction between SAP102 and the regulatory protein JNK3: when the full-length GluK2 C terminus is expressed together with SAP102 and JNK3, the binding of JNK3 to SAP102 is clear, whereas when we delete the PDZ-binding motif of GluK2, the interaction between JNK3 and SAP102 is comparably weak (see Fig. 5*Ci* with quantification in Fig. 5*Cii*). We propose that binding of GluK2 to SAP102, which may be enhanced under specific physiological conditions, facilitates positive regulation by JNK and subsequent shuttling of GluK2 to the surface, with the assistance of the highly mobile SAP102. The analysis of targeted GluK2 mutants— namely, phospho-mimicking GluK2 point mutants that have been shown to remain internalized (30)—provided insights into the physiological conditions that might favor such regulation by JNK. Such recombinant GluK2 C termini (see schematic in Fig. 5*A*) display an increased binding affinity for JNK3 (see Fig. 5*Di* with quantification in Fig. 5*Dii*), suggesting that internalized GluK2 molecules might be especially available for JNK-mediated regulation, as one would expect if there was an acute physiological requirement for KARs to integrate into the synaptic membrane.

Our physiological observations in primary hippocampal neurons, together with our biochemical and pharmacological analysis in heterologous cells, strongly support the idea that JNK3, SAP102, and GluK2 are proteins that cooperate in the neuronal environment, and that formation of this multiprotein complex occurs in a controlled manner in response to specific signals, thus participating in the fine-tuned regulation of kainate receptor trafficking to the membrane.

## Discussion

**Figure 6:**
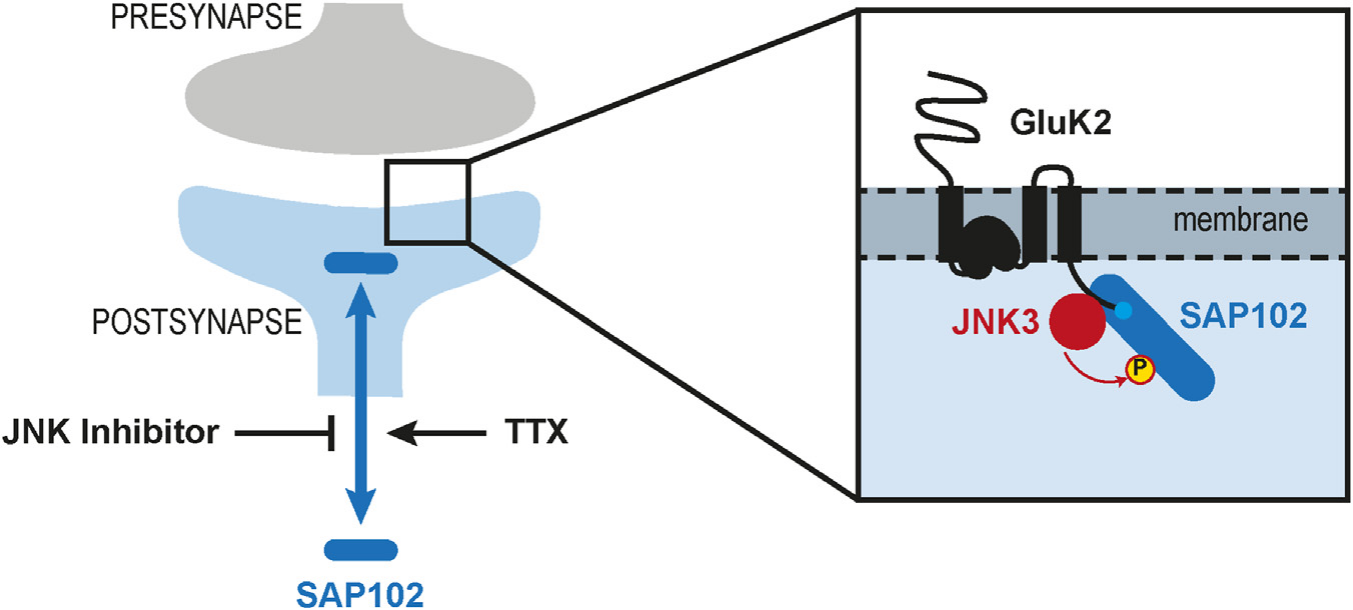
**Model illustrating the functional interplay between JNK, kainate receptors, and SAP102.** JNK modulates kainate receptor surface expression *via* phosphorylation-dependent regulation of SAP102 mobility.

In this study, we built on our previous observation that the postsynaptic scaffold protein SAP102 is biochemically associated with the regulatory kinase JNK3 (20). Compared to the related synaptic MAGUK scaffold molecule PSD-95, which is anchored at the PSD of glutamatergic synapses *via* palmitoylation of its N terminus (34), SAP102, which is not palmitoylated, moves into and out of dendritic spines with relative flexibility (17). This suggests that it may play a unique role in trafficking receptors to and from the PSD in response to physiological cues. We show here that this mobility can be negatively regulated by JNK inhibition under certain conditions and that SAP102 is indeed a direct phosphorylation target of JNK3. We further demonstrate that SAP102 and JNK3 are able to form a tripartite complex together with the ionotropic glutamate receptor GluK2. In line with a cooperative role for SAP102 and JNK3 in the regulated trafficking of this receptor, inhibiting JNK activity results in reduced GluK2 surface expression in cultured hippocampal neurons, which provides support for a model in which JNK-mediated regulation of SAP102 influences the trafficking of kainate receptors to postsynaptic sites (see model depicted in Fig. 6). Moreover, we demonstrate that the phospho-deficient SAP102 mutant, which cannot be phosphorylated by JNK, is less able than the WT to promote the surface expression of GluK2, providing further evidence supporting a role for JNK-mediated regulation of SAP102 in coordinating GluK2 trafficking to the membrane.

It has been shown that SAP102, like PSD-95 and other synaptic MAGUKs, is of critical importance for regulating glutamate receptor trafficking to synaptic sites (*e.g.*, (35)), and the consequences of MAGUK loss are especially apparent during synaptogenesis and synapse maturation (36), presumably in response to defective trafficking and anchoring of glutamate receptors and other PDZ-binding transmembrane proteins at the PSD. It has been clearly established that protein composition of the PSD of glutamatergic synapses is important for normal network development (37, 38), and MAGUK proteins are central hubs within this scaffold; factors that affect their functional integrity and proper regulation are thus obvious targets for investigation. Interestingly, it has been shown that diverse posttranslational modifications are indeed able to influence MAGUK function and resulting protein composition of the PSD (6, 19, 26, 39).

In this context, our observation that JNK activity modulates SAP102 mobility is of special relevance. JNK proteins are historically known for regulation of the cellular stress response and, in neurons, for modulating excitotoxicity. More recent studies have highlighted their importance in the regulation of normal physiological processes during neural development (for review see (22)). By modulating the motion of SAP102 into and out of dendritic spines, JNK signaling can potentially fine-tune the cellular localization of this scaffold and its binding partners, which inevitably influences the protein composition of PSDs at glutamatergic synapses. Importantly, our observation that JNK inhibition reduces kainate receptor surface expression reveals one of the many downstream effects that could result from such hindered SAP102 mobility.

The kainate receptor GluK2, like JNK3 and SAP102, has been implicated in brain function through genetic studies in humans and mice (29, 40, 41, 42). In our experimental setup, reduced JNK activity corresponds to reduced KAR expression at the membrane, suggesting that JNK signaling could play a role in regulating either the membrane trafficking GluK2 or its maintenance within the membrane. This role for JNK signaling provides a putative mechanistic explanation for the common phenotype of the GluK2 and JNK3 KO mouse models: both are resistant to kainate-induced seizures (43, 44). Moreover, our data, in which we highlight functional and biochemical links between both of these proteins and the synaptic scaffold SAP102, support the idea that these three molecules participate in a common signal cascade coordinating the cellular response during kainate-induced excitotoxicity and in the regulation of kainate-receptor function under normal physiological conditions.

Indeed, the three proteins are able to form a complex, and our biochemical experiments suggest that the simultaneous presence of the three proteins positively influences their individual affinities for one another: specifically, we observe an increased interaction stability between SAP102 and JNK3 in the presence of the PDZ-binding ligand GluK2 (see Fig. 5*C*). This result, which supports the idea that these three proteins indeed cooperate functionally, is in line with studies on macromolecular complex formation dictated by other MAGUKs: complex formation induced by ligand binding to PSD-95 PDZ domains has been demonstrated in both *in vitro* and cellular contexts (45, 46, 47, 48). Moreover, it has been demonstrated that C-terminal cytosolic tail of GluK2 binds directly to the PDZ domains of related synaptic MAGUKs and that these interactions have direct implications for kainate receptor function at synaptic sites (33, 49).

Finally, our data provide support for the idea that JNK3 participates in the regulated control of kainate receptor cycling to and from the postsynaptic membrane. We highlight that JNK inhibition leads to reduced KAR surface expression and that normal JNK activity levels correspond to higher KAR surface expression. We also observe that JNK3 binds favorably to GluK2 variants mimicking those that are internalized in response to PKC-mediated phosphorylation (30, 50, 51), in line with a regulatory action of JNK3 that is influenced by the cellular environment and the state of GluK2. We propose a functional model that reflects these data (see Fig. 6): in response to elevated synaptic activity, kainate receptors are posttranslationally modified by other kinases—including, for example, PKC—and receptor internalization is facilitated (52, 53), which prevents KAR hyperactivation and excitotoxicity. JNK3 binds preferentially to such internalized variants (Fig. 5*D*), perhaps in order to restore surface expression of synaptic KARs and maintain activity in a homeostatic process. Such a model depicts a positive role for JNK in modulating neuronal activity and likewise illuminates a putative mechanism for the documented resistance to kainate-induced seizures observed in both the JNK3 and GluK2 KO mice. Our data also support the idea that JNK3 executes its regulatory control of KAR function in cooperation with SAP102, which becomes more mobile when JNK is active or under conditions of reduced synaptic activity (see Figs. 1 and 4). Our model thus highlights how these three proteins can participate in a common signaling pathway, highlighting a putative mechanistic explanation for some of the common behavioral and synaptic transmission aberrations found in the corresponding disease models.

## Experimental procedures

### Antibodies

Antibodies used in this study include FLAG (mouse, Sigma F1804), FLAG-HRP (Sigma A8592, WB 1:1000–4000), GFP (mouse, Roche, 11814460001; goat, Abcam, AB6673, WB 1:4000; chicken, Abcam, ab13970, IF 1:5000–7500, WB 1:5000), glutathione-*S*-transferase (GST) (goat, GE Healthcare, 27457701V, WB 1:5000), MYC (mouse, Clontech, 631206; rabbit, Cell Signaling, 2272S, WB 1:5000), V5 (rabbit, Millipore, AB3792, WB 1:5000), and those directed against the following proteins: actin (rabbit, Sigma, A2066, WB 1 : 2000), GFAP (mouse, antibodies Inc, 75–240, WB 1:2000), GluK2/3 (rabbit, Millipore, 04-921, WB 1:4000, IF 1:500), Homer (guinea pig, Synaptic Systems, 160004, IF 1:500), JNK3 (rabbit, Millipore, 04-893, IF: 1:250), MAP2 (mouse, IF 1:500; guinea pig, Synaptic Systems, 188004, IF 1:1000), SAP102 (mouse, antibodies Inc, 75-058, WB 1:1000–5000, IF 1:100), SAP102 (rabbit, Abcam, ab3438, IF 1:500), tubulin (rat, Abcam, ab6160, WB 1:12000).

For Co-IP experiments, 2 μg of the respective antibody was used. Unspecific mouse IgGs, as required (SantaCruz, SC-2025, 2 μg), were used for negative controls in Co-IP studies. All primary and secondary antibodies were diluted in 5% milk/PBS with 0.1% Tween 20 (PBST) for WB or in 4% bovine serum albumin (BSA)/PBS for IF experiments. For WB experiments, we used the following secondary antibodies: α-mouse-HRP (Dianova #115035003 or α-native-mouse-IgG-HRP Abcam, ab131368), α-rabbit-HRP (Dianova #111-035-003), or α-goat-HRP (Santa Cruz #sc-2020) with a dilution of 1:5000. α-rat-HRP (Santa Cruz #sc-2032) and α-chicken-HRP (Abcam #ab6753) were used with a dilution of 1:10,000. For IF experiments, we used secondary antibodies α-guinea-pig-Alexa405 (Abcam, ab175678), α-chicken-Alexa488 (Dianova #703-545-155), and the following antibodies from Thermo Fisher Scientific: α-mouse-Alexa405 (A-31553), α-rabbit-Alexa405 (A-31556), α-rabbit-Alexa488 (A-21441), α-guineapig-Alexa568 (A-11075), α-mouse-Alexa568 (A-11031), α-rabbit-Alexa568 (A-11036), all with a dilution of 1:1000. For on-cell Western experiments, we used α-mouse-680RD (1:500, Li-COR #926-68070) and α-mouse-800CW (1:500, Li-COR #925-32218).

### Antibody against phosphorylated-S368-SAP102

The phosphorylation site S368 of SAP102 lies in the linker region between PDZ2 and PDZ3 of human SAP102 (DLG3_HUMAN, Q92796, 817 AA; OMIM #300850); S368 of human SAP102 corresponds to position S386 in rat SAP102 (DLG3_RAT, Q62936, 849 AA). Customized polyclonal phospho-specific antibodies were raised in rabbit against a phospho-peptide, including pS368 (DLG3_HUMAN, AA 361–373: QVPPTRY-S*(PO3H2)*-PIPRH; EUROGENTEC) and affinity-purified (α-p-SAP102, rabbit, WB: 1: 600–1000).

### DNA constructs

The construct pEGFP-C1-JNK3a2 has been published previously (20) and was used as a template for cloning JNK3 into pBudCE4 under the control of the EF1a promotor to generate a C-terminal V5-tagged expression construct JNK3-V5 (pBudCE4-JNK3-V5). We used the pEGFP-C1-JNK3 as a template for cloning EGFP-JNK3 into the lentiviral shuttle vector f(syn)w (based on FUGW (54)) for expression under the control of a synapsin promotor in primary rat hippocampal neurons.

Phospho-deficient SAP102 variant (pCMV2A-FLAG-SAP102-S368A) was generated by site-directed mutagenesis using pCMV2A-FLAG-SAP102 (20) as a template. Full-length SAP102 was also cloned into pCMV3A to generate N-terminal MYC-tagged construct (pCMV3A-MYC-SAP102). For the generation of the SAP102 lentiviral expression construct, we cloned SAP102 into the pEGFP-N1 vector using pCMV2A-FLAG-SAP102 as a template. In a second step, we used the pEGFP-N1-SAP102 as a template for cloning SAP102-EGFP into the lentiviral shuttle vector f(syn)w. The N-terminal GST-SAP102-PDZ1-PDZ3 fusion construct (pGEX-6P-1-FLAG-SAP102-PDZ1-3) was subsequently generated from pCMV2A-FLAG-SAP102. This fragment, encoding an N-terminal FLAG tag and the first 472 amino acids of SAP102, was cloned into the pGEX-6P-1 vector with an N-terminal GST tag (GE Healthcare).

The construct pcDNA3-6xMYC-GluK2 was a gift from C. Mulle (Bordeaux) and was used as a template for the cloning of the cytoplasmic C-terminal tail of GluK2 (pEGFP-C1-GluK2-cyto; 841–908 aa of P39087, GRIK2_MOUSE), the shorter deletion construct of the cytoplasmic tail of GluK2 (pEGFP-C1-GluK2-cyto-short; 841–882 aa of P39087), and the generation of mutant variants by site-directed phospho-mimicking mutagenesis of S846E and S868E (pEGFP-C1-GluK2-cytoINT, pcDNA3-6xMYC-GluK2-INT).

### Cell culture

Heterologous cells that allow for robust expression of recombinant proteins in our hands, namely Chinese hamster lung cells (CHL-V79) and human embryonic kidney cells (HEK293T)—validated by PCR—were used for all cell-based experiments that were not carried out in primary neurons (see Figure legends for details on cell type used). Cells were maintained in Dulbecco's modified Eagle's medium supplemented with 10% FBS, 2 mM L-glutamine and penicillin/streptomycin in a humidified incubator at 37 °C with 5% CO_2_. Transfections were performed using Lipofectamine 2000 (Invitrogen) and Opti-MEM (Gibco) according to manufacturer’s instructions.

Primary rat E18 hippocampal neurons were prepared as already described (55), in accordance with the Directive 2010/63/EU of the European Parliament on the protection of animals used for scientific purposes. Protocols for animal sacrifice were approved by the Regional Office for Health and Social Affairs in Berlin (“Landesamt für Gesundheit und Soziales; LaGeSo”) and the animal welfare committee of the Charité and carried out under permits T0280/10 and T-CH 0002/21. Briefly, embryonic E18 Wistar rats were used for hippocampi isolation and dissociation. Dissociated cultures were plated in Neurobasal medium, infected at DIV10 with lentivirus and harvested, treated, or analyzed after 3 weeks in culture between DIV20 and DIV24. Treatments of neurons with JNK inhibitor SP600125 (25 μM) or TTX (2 μM) were done in conditioned medium.

### IF and confocal microscopy

IF experiments were performed as described previously (20) according to standard IF protocols. Dissociated cultures of primary rat hippocampal neurons were fixed in 4% paraformaldehyde (PFA) in PBS for 10 min, washed in PBS, permeabilized in 0.2% Triton-X in PBS for 5 min, washed in PBS, and blocked in 4% BSA in PBS for 1 h at room temperature. Cells were incubated with primary antibodies overnight at 4 °C in blocking solution, washed with PBS, incubated with secondary antibodies for 30 to 60 min in blocking solution, and finally washed in PBS. Coverslips were mounted on glass slides with Fluoromount-G (Southern Biotech). Antibodies used for IF experiments were either established commercially available antibodies for important neuronal marker proteins or tested for specificity on overexpressed proteins in our hands.

Cells were imaged using a confocal laser scanning microscope (TCS-SP5 II, Leica). Images were acquired with a 63× objective (1.5–2× zoom, 1024 × 1024 px, 0.4 μm steps in z, z-planes). Images shown are single z-planes with the strongest signal intensity in the region of interest.

### Live-cell imaging: FRAP

Primary rat E18 hippocampal neurons plated in FluoroDishes (cover-glass bottom, 35 mm, WPI Inc) were infected at DIV10 and analyzed in live-cell experiments at DIV20-24. Neurobasal medium was exchanged with Tyrode solution (25 mM Hepes pH 7.4, 120 mM NaCl, 2.5 mM KCl, 2 mM CaCl_2_, 2 mM MgCl_2_, 30 mM glucose) or extracellular solution (ECS: 25 mM Hepes pH 7.4, 140 mM NaCl, 5.4 mM KCl, 1.3 mM CaCl_2_, 33 mM glucose) 30 min before measurement. In case of treatment with SP600125 or TTX, conditioned medium including the drugs was exchanged with solution including the respective treatment.

FRAP experiments were performed using a NIKON spinning disc confocal CSU-X microscope (FRAP/PA, 60× Plan Apo objective with NA = 1.4, laser: 488 nm, emission filter: 525/50, Andor DU-888 X-9798 camera, Perfect Autofocus PFS, NIS Software, https://www.microscope.healthcare.nikon.com/en_EU/products/software). FluoroDishes were acclimated in the live-cell chamber of the microscope for 20 min (37 °C, 5% CO_2_) before starting the measurement. Spines (10–15 spines per image) of SAP102-EGFP–expressing neurons were selected as regions of interest (ROIs) for bleaching and imaging: ten prebleach images, photo-bleaching (laser 488 nm 100%, dwell time 100 μs, 15× bleaches), followed by 20 acquisitions with an interval of 3 s and further followed by 30 acquisitions with an interval of 30 s (postbleach images: laser 488 nm 20%, 300 ms exposure time, 538 × 538 px, total duration of FRAP measurement 960 s). Three FRAP acquisitions per dish have been performed sequentially.

Images have been analyzed using NIKON NIS Software for ROI inspection over time, drift correction in X/Y, fluorescence intensity measurements, and background subtraction. Spines, which disappeared during measurement, were excluded from further analysis. ROIs of highly moving spines were manually adjusted. For the FRAP calculation, the first three of ten prebleach images were deleted (56), and the fluorescence values for each ROI over time were background-corrected and normalized (EXCEL): the mean of the last seven of ten prebleach values was set to 1 (100%), whereas the lowest intensity value (first postbleach image, t = 0) was set to 0 (0%). All other intensity values were normalized accordingly for each ROI. We measured per condition (control, TTX, SP600125, TTX+SP600125, Fig. 4) a total of 94 to 112 spines (10–15 spines per image; 2–4 images per FluoroDish; three FluoroDishes from three different neuronal cultures) and calculated the mean and the SEM. The mobile fraction was calculated as the mean of the last eight FRAP values of all ROIs for each condition. The data passed tests for normal distribution (D’Agostino & Pearson test, Shapiro–Wilk test and Anderson–Darling test). Statistical significance was determined by performing one-way ANOVA, followed by Dunnett’s multiple comparisons testing or by unpaired two-tailed *t* test. For the statistical analysis, Graphpad PRISM 10 software was used. The t_1/2_ (half-time of recovery) as the half maximal recovery time of the mobile fraction of SAP102-EGFP was estimated on the basis of the FRAP graph.

### Coimmunoprecipitation

Transfected cells (CHL V79 or human embryonic kidney 293T) were harvested 20 to 45 h after transfection and resuspended in lysis buffer (50 mM Tris–HCl pH 7.5, 100 mM NaCl, 0.1% Triton-X (or 1% Triton-X for co-IPs with full-length MYC-GluK2, Fig. 5*Bi*), including protease inhibitors (Mini Complete without EDTA, Roche)). After lysis using a 30-gauge syringe and 30 min incubation on ice, lysates were centrifuged twice (10 min, 20,000*g*, 4 °C), and the resulting supernatant was incubated with 2 μg antibody or normal mouse IgG for 3 h at 4 °C in a spinning wheel. To remove precipitated proteins, the lysates were centrifuged (10 min, 20,000*g*, 4 °C), and the resulting supernatants were added to 25 μl Protein G Agarose (Roche), rotating for 1 h at 4 °C, followed by three times washing (each 5–10 min) with lysis buffer. Analysis of precipitated were analyzed by Western blotting.

### *In vitro* kinase assay

The nonradioactive, *in vitro* kinase assay was performed as described before (20). Briefly, bacterially expressed GST-SAP102-PDZ1-3 was purified, eluted, desalted (55), and subsequently used as a substrate for the phosphorylation by JNK3 (commercially available from BioMol). Reactions were performed in 20 mM Tris pH 7.5, 10 mM MgCl_2_, 200 μM ATP, 0.1% beta-mercaptoethanol for 30 min at 30 °C. SAP102 phosphorylation was analyzed by Western blotting with α-p-SAP102.

### Surface biotinylation

Primary rat hippocampal neurons (DIV20-24, in 6 well) were washed with PBS on ice and then biotinylated for 30 min (1 mg/ml EZ-Link Sulfo-NHS-LC-Biotin, Thermo Fisher Scientific, in PBS) on ice. After three PBS washing steps, the biotinylation reaction was quenched with 100 mM glycine/PBS (2 × 5 min) and washed again twice in PBS on ice. Cells were harvested by scraping in lysis buffer (50 mM Tris–HCl pH 7.5, 100 mM NaCl, 1% Triton-X, 0.1% SDS, 0.5% sodium deoxycholate including Mini Complete Inhibitors (Roche)) and lysed on ice for 30 min after sonication. Cleared lysate (twice centrifugation for 10 min at 20,000*g*) was incubated for 3 h with 50 μl Dynabeads M-280 Streptavidin (Invitrogen), followed by three washing steps with lysis buffer. Biotinylated proteins were analyzed by Western blotting.

### Western blot and Phos-tag

Protein samples for Western blotting were boiled at 95 °C for 5 min in 2× SDS sample buffer and separated on standard Lämmli SDS-PAGE gels. Proteins were semidry blotted onto polyvinylidene fluoride (PVDF) membranes (Roche), which were subsequently blocked in 5% milk in PBST, incubated with primary antibodies in 5% milk in PBST over night at 4 °C, followed by three PBST washes, incubation with the secondary antibodies for 1 h in 5% milk in PBST and final three washes with PBST. Chemiluminescence signals were detected using the imager ImageQuant LAS4000mini (GE Healthcare) or IQ800 (Cytiva) or a film developer machine.

Phos-tag (WAKO) gel electrophoresis for analysis of phosphorylated SAP102 as well as the sample preparation for phosphatase treatment was done as described before (55) using Bis-Tris–buffered neutral pH gels with 6% polyacrylamide supplemented with 20 to 75 μM Phos-tag.

### WB image analysis

For the quantification of WBs, we used the software ImageQuantTL v10 (Cytiva) (https://www.cytivalifesciences.com/en/de/shop/protein-analysis/molecular-imaging-for-proteins/imaging-software/imagequant-tl-10-2-analysis-software-p-28619). After lane selection, background was subtracted using the rubber band subtraction method. Bands of interest were automatically detected (automatic edge mode) and then manually adapted for blot internal comparable measurements (band size). Statistical significance was determined by performing one-way ANOVA, followed by Dunnett’s multiple comparisons testing or by unpaired two-tailed *t* test. The data passed test for normal distribution (Shapiro–Wilk normality test). For the statistical analysis, GraphPad Prism 10 software (https://www.graphpad.com/) was used.

### On-cell Western

GluK2 surface expression experiments were performed using the cell-based on-cell Western assay, which enabled the quantitative detection of surface expressed MYC-tagged GluK2 in heterologous CHL-V79 cells by an IF staining procedure (near-IR fluorescence), followed by acquisition using the LI-COR Odyssey CLx Imager. Transfected CHL cells in multiwell plates were carefully washed in PBS, fixed in 4% PFA-PBS for 10 min, washed in PBS, blocked for 30 min in BSA-PBS, incubated with α-MYC (mouse, 1:500, Clontech #631206) in 4% BSA-PBS for 60 min at room temperature to stain surface expressed GluK2, washed in PBS, incubated with α-mouse-800CW (1:1000, LI-COR #925-32218) for 30 min, and washed in PBS (surface staining completed), followed by a standard IF protocol: samples were fixed in 4% PFA-PBS for 10 min, washed in PBS, permeabilized for 5 min in 0.2% Triton-X-PBS, washed in PBS, blocked in 4% BSA-PBS for 30 min, incubated with α-MYC (mouse, 1:1000) over night at 4 °C, washed in PBS, and incubated with α-mouse-680RD (1:1000, LI-COR #926-68070) for 30 min, followed by PBS wash.

Experiments were performed in sextets (six transfected multiwells per condition): three of the six wells were used for surface (800 nm) and total (700 nm) staining of MYC-GluK2 (triplicates), whereas the other three wells were used for controls of FLAG-SAP102 expression (800 nm) and cell density (700 nm). The staining procedure for the SAP102 expression and cell density control was performed as described above, but the surface staining steps were omitted. FLAG-SAP102 proteins were stained using α-FLAG (mouse, 1:1000) and α-mouse-800CW (1:1000), cell density was controlled by using CellTag700 (LI-COR, #926-41090) during the incubation of the secondary antibodies according to manufacturer’s recommendations.

The multiwell plates were imaged using the LI-COR Odyssey CLx Imager and further analyzed by using the software Image Studio (https://www.licor.com/bio/image-studio/) (LI-COR Biosciences), FIJI (https://imagej.net/software/fiji/) (57) (including the plugin Readplate2.1 (58)), EXCEL (Microsoft), and Prism 10 (GraphPad). A grid was used to identify the wells; the mean gray values were measured for images at 700 and 800 nm, followed by a background subtraction for each channel. The ratio of the 800 nm/700 nm intensities (Surface GluK2 fraction/total GluK2) was calculated for each well. As a control for nonsurface GluK2 expression, we used the expression of an internalized GluK2 receptor variant (MYC-GluK2-INT with phospho-mimicking mutation at S846E and S868E). The 800/700 ratio (mean of triplicates) of the MYC-GluK2-INT samples was subtracted (set to 0) from all single wells, followed by a normalization to the mean of the triplicates of MYC-GluK2 surface (800)/total (700) staining (set to 1). Three to five independent experiments were performed, each including technical replicates. Statistical significance was determined by performing unpaired two-tailed *t* test.

## Data availability

The data supporting the findings of the study are available in the manuscript and Supplementary information. Other raw data generated in the study are available from the corresponding author on reasonable request.

## Supporting information

This article contains supporting information.

## Conflict of interest

The authors declare that they have no conflicts of interest with the contents of this article.
