# Supplementary material for: JNK activity modulates postsynaptic scaffold protein SAP102 and kainate receptor dynamics in dendritic spines
Source: J Biol Chem. 2024 Apr 4;300(5):107263. doi: 10.1016/j.jbc.2024.107263 (PMC11081805; doi:10.1016/j.jbc.2024.107263)
Supplement: Supporting Figures S1–S6 [file mmc1.pdf]

JNK activity modulates postsynaptic scaffold protein SAP102 and kainate receptor dynamics in dendritic spines

Stella-Amrei Kunde, Bettina Schmerl, Judith von Sivers, Elham Ahmadyar, Taanisha Gupta, Nils Rademacher, Hanna L. Zieger and Sarah A. Shoichet

Supplementary Figure S1

i

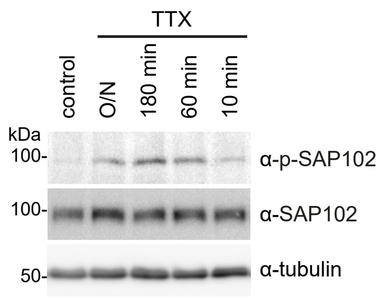

ii

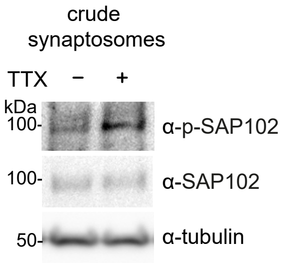

Endogenous neuronal SAP102 is phosphorylated after TTX treatment.
i) Western blot analysis of SAP102 phosphorylation in primary rat hippocampal neurons with 2 μM TTX treatment for different durations (O/N, 180 min, 60 min, 10 min); α-tubulin serves as a loading control (n=3).
ii) Detection of phosphorylated SAP102 after TTX treatment (2 μM, O/N) in crude synaptosome preparations from primary rat hippocampal neurons (DIV23), n=4.

Supplementary Figure S2

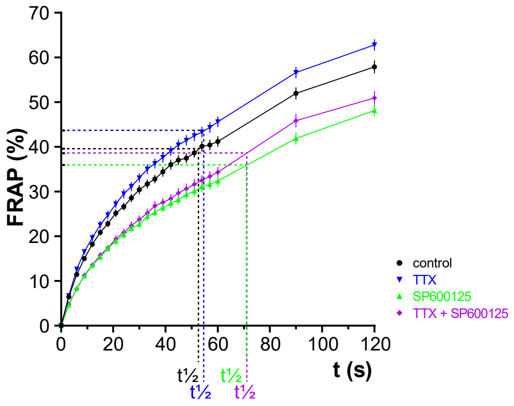

FRAP experiments of SAP102-EGFP in hippocampal neurons: mobile fraction (Fig 4Aii) was used for estimation of half-time of recovery (t1/2) of mobile SAP102-EGFP, based on the FRAP experiments shown in Fig 4Ai.

Supplementary Figure S3

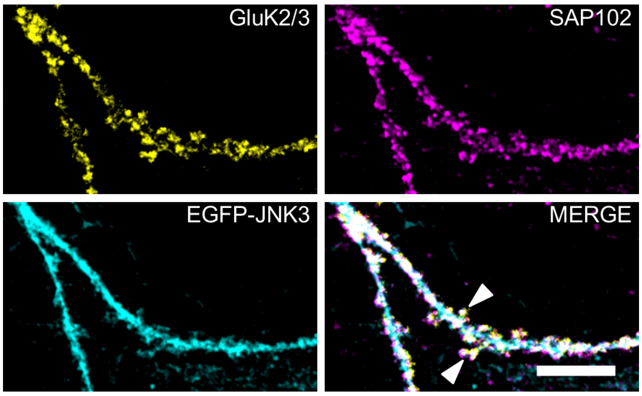

Co-staining of endogenous GluK2/3 (yellow, Alexa405), SAP102 (magenta, Alexa568) and EGFP-JNK3 (cyan, EGFP/Alexa488) in rat hippocampal neurons (DIV22). Arrowheads show partial co-localisation of all three proteins in spines (MERGE). Scale bar: 10 μm.

Supplementary Figure S4

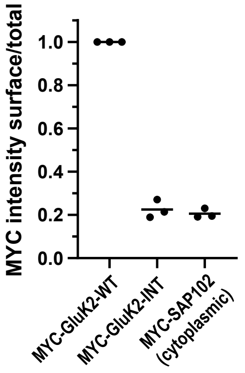

On-cell Western (OCW) assay for surface expression of overexpressed MYC-GluK2 in transfected CHL cells. Normalised MYC surface fraction/total staining for MYC-GluK2-WT, MYC-GluK2-INT (internalised GluK2) and MYC-SAP102 (control: only cytoplasmic expression). Data show mean of three independent experiments (n=3) each consisting of technical replicates.

Supplementary Figure S5

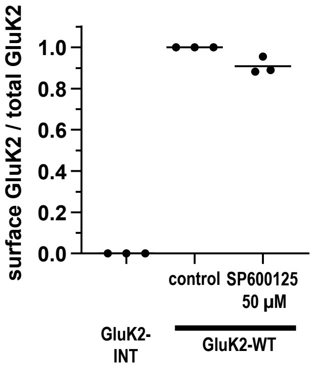

Analysis of GluK2 surface expression (relative to total GluK2 expression) following expression in CHL cells (On-Cell Western, OCW) together with wild-type SAP102 indicates that the relative GluK2 surface expression decreases with JNK inhibitor SP600125 (50 µM). Data are mean of three biological replicates (n=3) each consisting of technical replicates.

Supplementary Figure S6

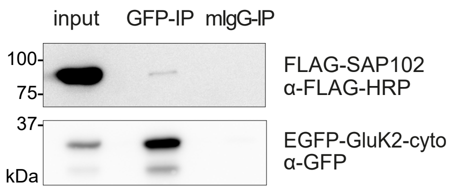

SAP102 interacts with the cytoplasmic GluK2-C-terminus in co-immunoprecipitation experiments (HEK293T). Pulldown of overexpressed EGFP-GluK2-cyto (GFP-IP) showed co-precipitation of FLAG-SAP102 compared to mIgG-IP (pulldown with unspecific mIgGs as negative control). n=3.
